# Selenium nanoparticles alleviate cobalt toxicity in artificial joint metal prostheses by inhibiting ferroptosis through activation of the PRDX6/GPX4 pathway

**DOI:** 10.1016/j.mtbio.2025.102306

**Published:** 2025-09-12

**Authors:** Pengcheng Xu, Fan Liu, Su Jiang, Baisheng Cai, Cong Ye, Yiming Sun, Yaping Wang, Jining Shen, Huan Zhou, Yake Liu

**Affiliations:** a Department of Orthopaedics, Affiliated Hospital of Nantong University, Nantong, 226001, China; b Medical School of Nantong University, Nantong, 226001, China; c Center for Health Science and Engineering, Hebei Key Laboratory of Biomaterials and Smart Theranostics, School of Health Sciences and Biomedical Engineering, Hebei University of Technology, Tianjin, 300131, China

**Keywords:** Cobalt-containing metal implants, Cobalt nanoparticles, Cobalt toxicity, Ferroptosis, Selenium nanoparticles

## Abstract

The long-term implantation of metallic joint prostheses results in the release of cobalt nanoparticles (CoNPs), leading to local and even systemic toxic reactions that pose risks to patient health. Previous studies have suggested that CoNPs-induced cytotoxicity may be associated with excessive oxidative stress and ferroptosis. Selenium nanoparticles (SeNPs), known for their anti-ferroptotic properties, have potential as surface coatings for metal implants. This study aims to investigate the role and mechanisms of SeNPs in inhibiting ferroptosis and mitigating cobalt-induced toxicity, thereby offering a mechanistic rationale for improving the material properties of metal prostheses. We first conducted molecular analyses on tissue samples from patients undergoing hip joint revision surgery, which revealed activation of ferroptosis-related signaling pathways. We then synthesized SeNPs capable of effective internalization by bone marrow-derived stromal cells (BMSCs). In vitro, 400 μM CoNPs induced hallmark features of ferroptosis in BMSCs by suppressing the SLC7A11/GPX4 axis and activating the HIF-1α/HO-1 signaling pathway. In contrast, treatment with 40 μM SeNPs upregulated PRDX6 and GPX4, thereby attenuating ferroptosis and preserving cell viability. Finally, intra-articular injection of SeNPs into mouse knee joints significantly alleviated CoNPs-induced local toxic responses, including synovial hyperplasia and cartilage destruction. Overall, this study provides novel insights into the ferroptosis-dependent mechanisms underlying CoNPs-induced toxicity and highlights the therapeutic potential of SeNPs as a detoxifying agent. These findings offer a mechanistic foundation for targeted detoxification strategies and inform the development of improved metal prosthetic materials.

## Highlights

• CoNPs induce cytotoxicity through a non-apoptotic cell death mechanism.
• Ferroptosis is strongly associated with cobalt toxicity caused by cobalt-based alloy implants *in vivo.*
• CoNPs promote ferroptosis via the SLC7A11/GPX4/HIF-1α/HO-1 pathway, contributing to their toxic effects.
• SeNPs upregulate PRDX6, enhancing the defense against ferroptosis.
• SeNPs provide a novel strategy for active and targeted detoxification of cobalt toxicity at its source.

## Graphical abstract

**Figure:**
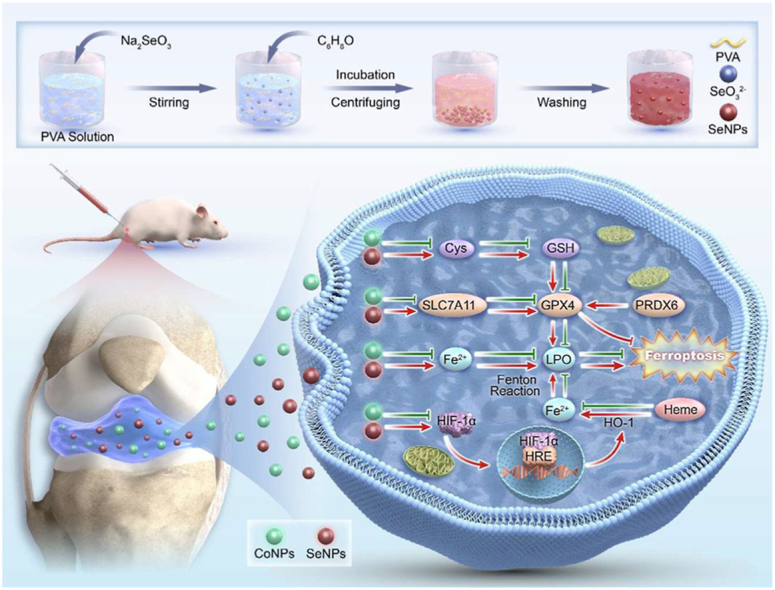

## 1 Introduction

Cobalt-based alloys are commonly used in clinical practice for metal implants in total hip arthroplasty (THA) and total knee arthroplasty (TKA) due to their superior mechanical properties, corrosion resistance, wear resistance, and biocompatibility [[1], [2], [3]]. However, after implantation, these alloys experience wear over time due to various physicochemical factors, including fatigue micromotion, and mechanical stress [4], leading to the release of cobalt nanoparticles (CoNPs), which accumulate around the implant and lead to local toxic reactions, including inflammation, bone resorption, and implant loosening [5,6]. Furthermore, these particles enter the systemic circulation, causing chronic, insidious, and progressively worsening toxicity. This is particularly prominent in metal-on-metal (MOM) THA [4,7], which has been recognized as a unique complication of these implants, ultimately leading to their removal from clinical use. In addition, other friction interfaces, such as metal–polyethylene and ceramic–ceramic prostheses, may also exhibit signs of cobalt toxicity, including inflammatory pseudotumors and the accumulation of metal particles during revision surgeries [8,9].

The toxic mechanisms of CoNPs are complex, involving oxidative stress, inflammatory responses, and cell apoptosis [10,11]. Recent studies have shown that cobalt exposure significantly increases iron accumulation in tissues [12]. Unlike apoptosis, necrosis, and autophagy, ferroptosis is a newly discovered iron-dependent form of regulated cell death [13]. Our previous research demonstrated that CoNPs induce dose-dependent cell death in BALB/3T3 cells through a non-apoptotic mechanism, this process is accompanied by increased levels of intracellular reactive oxygen species (ROS) and cytoplasmic Fe^2+^, along with a reduction in reduced glutathione (GSH) [14]. These findings align with the biochemical characteristics of ferroptosis. Moreover, the addition of natural antioxidants and ferroptosis inhibitors alleviates CoNPs-induced cytotoxicity [14,15], suggesting that the toxicity of CoNPs may be closely linked to excessive oxidative stress and ferroptosis. Further investigation into ferroptosis-mediated CoNPs toxicity and the development of effective detoxification strategies are crucial.

Selenium is an essential trace element crucial for metabolism and cellular functions [16]. It is incorporated into selenoproteins, which are key components of antioxidant enzymes involved in free radical scavenging and antioxidant defense [17,18]. Selenium compounds include inorganic selenium, organic selenium, and selenium nanoparticles (SeNPs). SeNPs, with their nanometer size, offer advantages such as greater stability, higher bioavailability, and lower toxicity [16]. Notably, SeNPs possess enhanced antioxidant properties, enabling them to scavenge free radicals and boost the activity of antioxidant selenoenzymes [18,19]. Our research demonstrated that selenomethionine (Se-Met) protects hematopoietic cells from CoNPs-induced toxicity by enhancing antioxidant capacity and activating DNA damage response signaling [20]. Recent studies have shown that SeNPs inhibit ferroptosis by enhancing glutathione peroxidase 4 (GPX4) expression [21,22], alleviating brain ischemia-reperfusion injury [23], acute kidney injury [24], and acute lung injury [25]. Additionally, Selenium also detoxifies heavy metals like cadmium, mercury, and silver [26,27]. Given the anti-ferroptotic bioactivity of SeNPs and their potential for surface modification through nanocoating technology on metal implants [28], SeNPs show promise in mitigating cobalt toxicity in artificial joint metal prostheses by inhibiting ferroptosis, thus providing a theoretical foundation for the development of selenium-coated joint prostheses.

**Fig. 1:**
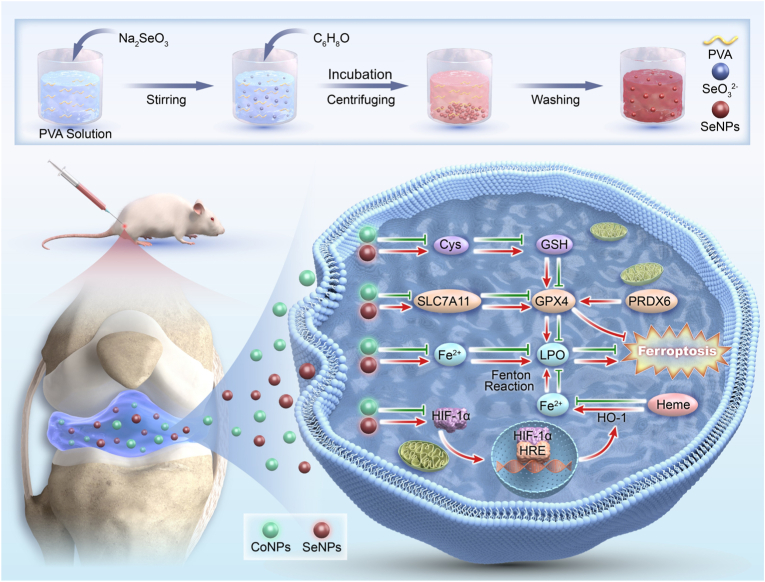
Schematic diagram illustrating the chemical reduction method for the preparation of SeNPs and their potential mechanism in inhibiting ferroptosis to counteract cobaltism.

In this study, we explored the link between local toxic responses of cobalt-based metal implants and ferroptosis by collecting synovial tissue and inflammatory pseudotumors from hip revision surgeries for molecular analysis. Additionally, SeNPs were synthesized using sodium selenite, ascorbic acid, and PVA. *In vitro* studies analyzed ferroptosis in CoNPs-induced toxicity and SeNPs-mediated detoxification. Finally, CoNPs and SeNPs were injected into mouse knee joints to investigate *in vivo* detoxification mechanisms. The complete experimental procedure is outlined in the following schematic (Fig. 1).

## 2 Methods and material

### 2.1 RNA-seq and bioinformatic analysis of human specimens

The experimental procedures involving human specimens were reviewed and approved by the Ethics Committee of the Affiliated Hospital of Nantong University (Approval No. S20240927-008). RNA-seq analysis was performed on tissue samples collected from the surrounding areas of hip joint replacement prostheses. Total RNA was extracted for identification, and after confirming the quality of the library construction, sequencing was performed using the Illumina high-throughput sequencing platform with a PE150 sequencing strategy. Differential expression analysis was conducted using the DESeq2 R software between two comparison groups (each group containing 4 biological replicates). The criteria for differentially expressed genes were: fold change ≥1.5, *p* < 0.05, and *q* < 1. Kyoto Encyclopedia of Genes and Genomes (KEGG) pathway enrichment analysis of differentially expressed genes was carried out using the ClusterProfiler package, with a significance threshold set at *p* < 0.05.

### 2.2 Preparation of SeNPs

Sodium selenite (Na_2_SeO_3_), ascorbic acid (C_6_H_8_O_6_), and poly(vinyl alcohol) (PVA, degree of polymerization: 1750 ± 50, degree of hydrolysis: ≥99 %) were purchased from Sinopharm Chemical Reagent Co., Ltd. (China). Considering the influence of synthesis conditions on the bioactivity of SeNPs, a chemical reduction method, optimized based on previously reported protocols, was employed to prepare the SeNPs solution [29]. The detailed preparation procedure is as follows (Table S1): 0.3 g of PVA was dissolved in 15 mL of deionized water under stirring at 95 °C to form a PVA solution. At room temperature, Na_2_SeO_3_ was mixed with the PVA solution in a 1:1 vol ratio, followed by the addition of ascorbic acid solution at a volume ratio of 3:20 (PVA + Na_2_SeO_3_:C_6_H_8_O_6_). The mixture was stirred thoroughly using a magnetic stirrer and allowed to stand at room temperature for 2 h to ensure complete reduction of Na_2_SeO_3_ by C_6_H_8_O_6_, resulting in elemental selenium. The resulting solution was aged for 24 h, centrifuged at 9000 rpm for 10 min, and the red precipitate was washed twice before being dispersed in deionized water.

### 2.3 Characterization of SeNPs

The optical properties of the SeNPs suspension were first analyzed using a UV–Vis spectrophotometer (UV-2600i). Subsequently, the microscopic morphology of the SeNPs was observed using scanning electron microscopy (SEM). A droplet of SeNPs suspension was placed onto a carbon rod and air-dried for 15 min. SEM imaging was performed with an S4800 system (Hitachi) operating at 3 kV. Next, the particle size distribution of the SeNPs was determined by dynamic light scattering (DLS) using a Zetasizer Pro (Malvern Panalytical) after diluting the suspension. Simultaneously, the zeta potential of SeNPs suspensions was measured to assess their stability. The elemental composition of the nanoparticles was further analyzed using energy dispersive X-ray spectroscopy (EDX, JED-2300). X-ray diffraction (XRD) analysis was performed to detect the crystallographic phase composition of the nanoparticles within the 2θ range of 20°–80°. XRD (Malvern Panalytical) scans were conducted using Cu Kα radiation (λ = 1.5402 Å) with a scanning rate of 2° per minute and a step size of 0.02°. Finally, to determine the intracellular localization of SeNPs, BMSCs were co-cultured with 40 μM SeNPs for 24 h, following previously reported protocols [30]. Cells were then harvested, fixed, and processed for transmission electron microscopy (TEM, HT7800, Hitachi) to visualize the distribution of SeNPs within BMSCs. To complement imaging, EDX was performed for elemental composition analysis.

### 2.4 Isolation and culture of mouse bone marrow mesenchymal stem cells (BMSCs)

Bone marrow cells were collected from the femoral cavities of 4 w-old C57BL/6J mice (purchased from the Nantong University Experimental Animal Center). The cells were centrifuged, and the supernatant was discarded. Red blood cells were lysed using a red blood cell lysis buffer, and the remaining pellet was collected as primary cells. The cells were passaged when they reached 85 % confluence and subcultured at a 1:3 ratio. BMSCs were cultured in α-MEM medium supplemented with 1 % penicillin-streptomycin and 10 % fetal bovine serum in a cell incubator. All experiments were performed using third-generation BMSCs.

### 2.5 Cell viability assay

CoNPs (<50 nm) were purchased from Sigma-Aldrich. The selenium concentration in the SeNPs suspension was determined using inductively coupled plasma optical emission spectrometry (ICP-OES, Optima 8000/S10), with concentrations diluted to a range of 10∼80 μM. BMSCs were seeded into 96-well plates, and after complete cell adhesion, the medium was replaced. In the experimental groups, culture media containing different concentrations of nanoparticles were added, and cells were cultured for 1 and 3 d. After incubation, the medium was replaced with CCK-8 working solution (NCM Biotech, C6005), and cells were incubated for 2 h. Absorbance was measured at 450 nm to assess cell viability. Each group included five replicates, and the experiment was independently repeated three times.

### 2.6 Mitochondrial morphology analysis by TEM

BMSCs were seeded into 6-well plates and incubated until adhesion. Subsequently, nanoparticles were added, and the cells were further incubated for 24 h. After incubation, the cells were collected and washed. Following fixation, the samples underwent gradient ethanol dehydration, resin infiltration, embedding, and sectioning. The sections were stained with uranyl acetate and lead citrate before being imaged using a TEM. The TEM analysis focused on observing mitochondrial morphological changes. Quantitative analysis of mitochondria was performed using ImageJ software.

### 2.7 Total ROS assay and flow cytometric analysis

After co-incubation of BMSCs with nanoparticles for 24 h, cells were treated with 10 μM DCFH-DA (Beyotime, S0033S) and incubated at 37 °C in the dark for 20 min. Cells were then washed three times with serum-free culture medium to remove excess DCFH-DA. Fluorescence images were immediately captured using an inverted fluorescence microscope, and fluorescence levels of DCF were analyzed by flow cytometry. Data were analyzed using FlowJo software.

### 2.8 Lipid ROS assay

Lipid ROS levels in BMSCs after 24 h of nanoparticle treatment were assessed using the BODIPY 581/591 C11 dye (Thermo Fisher, D3861). Cells were incubated with 2 μM C11-BODIPY for 30 min. After adding 500 μL PBS to each well, fluorescence images were captured using a fluorescence microscope. The reduced form of the dye emits red fluorescence, while the oxidized form emits green fluorescence. The green/red fluorescence ratio was calculated using ImageJ software to represent the level of lipid peroxidation.

### 2.9 GSH/GSSH measurement

The GSH/GSSG ratio in BMSCs after 24 h of nanoparticle treatment was measured using the GSSG/GSH Quantification Kit II (DOJINDO, G263). Forty microliters of GSH/GSSG standard solution were added to each well and incubated for 1 h. Then, 60 μL of GSH/GSSG working solution was added, and absorbance was measured at 405 nm using a microplate reader. The concentrations of GSH and GSSG, as well as the GSH/GSSG ratio, were calculated based on the standard curve.

### 2.10 Determination of Fe^2+^ in mitochondria

Mito-FerroGreen (DOJINDO, M489) was used to detect Fe^2+^ levels in the mitochondria of BMSCs. After treatment with nanoparticles for 24 h, cells were incubated with 5 μM Mito-FerroGreen working solution for 30 min. Fluorescence images of stained cells were captured using an inverted fluorescence microscope, and fluorescence intensity in each group was quantified using ImageJ software.

### 2.11 Immunofluorescence of GPX4 in BMSCs

After treatment with nanoparticles, BMSCs were fixed with 4 % paraformaldehyde for 15 min, permeabilized with 0.1 % Triton X-100 for 5 min, and blocked with 5 % bovine serum albumin (BSA) for 1 h. The cells were then incubated overnight at 4 °C with the primary antibody against GPX4 (Abcam, ab125066; dilution 1:200). Alexa Fluor® 488-conjugated goat anti-rabbit secondary antibody (Servicebio, GB25303; dilution 1:500) was applied and incubated for 1 h in the dark. Subsequently, cells were stained with Cy5-labeled Cyclic Peptide (Servicebio, G1249) for 30 min and DAPI staining solution (Servicebio, G1012) for 10 min, both in the dark. Fluorescence images were captured using an inverted fluorescence microscope, and the green fluorescence intensity of each group was quantified using ImageJ software.

### 2.12 Quantitative Real-time PCR

After co-incubation of BMSCs with nanoparticles for 48 h, total RNA was extracted using TRIzol® Reagent (Thermo Fisher, 15596026CN). RNA was reverse transcribed to cDNA using 5X All-In-One RT MasterMix (abm, G486), and qRT-PCR was performed using EvaGreen 2X qPCR MasterMix (abm, MasterMix). Relative gene expression levels were calculated using the 2-ΔΔCt method, with fold changes compared to the control group. All primer sequences are listed in Table S2.

### 2.13 Western blotting

Protein samples from BMSCs co-cultured with nanoparticles for 48 h were extracted using RIPA buffer (NCM Biotech, WB3100). After determining the protein concentration, proteins were separated by SDS-PAGE using 10 % gradient gels (NCM Biotech, P40650) and transferred to a membrane at 400 mA. After blocking for 1 h, the membrane was incubated overnight at 4 °C with primary antibodies, including PRDX6 (Servicebio, GB113124; dilution 1:4000), SLC7A11 (Servicebio, GB115276; dilution 1:500), GPX4 (Abcam, ab125066; dilution 1:1000), HIF-1α (Abcam, ab308433; dilution 1:1000), and HO-1 (Abcam, ab189491; dilution 1:2000). After incubating with secondary antibodies at room temperature for 1 h, protein bands were captured using the Bio-Rad ChemiDoc Imaging System. The intensity of protein bands was quantified using ImageJ software, with β-actin serving as the internal loading control for all Western blot analyses.

### 2.14 Establishment of stable infected cell lines

Short hairpin RNA (shRNA) sequences were cloned into the pSIH1-H1-copGFP vector, while the full-length HIF-1α sequence was inserted into the phage-puro-FLAG vector. Lentiviral constructs including sh-PRDX6-1 (GCTTACAATGGTGAAACACCC), sh-PRDX6-2 (GGACGCTAACAACATGCCTGT), and a negative control shRNA (sh-NC) were synthesized and packaged by Gene Pharma (Shanghai, China). HEK-293T cells were transfected with the following lentiviral plasmids: phage-puro-FLAG (oe-NC), phage-puro-FLAG-HIF1α (oe-HIF-1α), pSIH1-H1-copGFP (sh-NC), pSIH1-H1-copGFP-PRDX6-1 (sh-PRDX6-1), and pSIH1-H1-copGFP-PRDX6-2 (sh-PRDX6-2). After 48 h, viral supernatants were harvested and filtered for downstream infection. BMSCs at the logarithmic growth phase were resuspended at a density of 5 × 10^4^ cells/mL and seeded into 6-well plates. After overnight incubation at 37 °C, cells were infected with the lentiviral supernatant at a multiplicity of infection (MOI) of approximately 1 × 10^8^ TU/ml. For knockdown constructs, transduction efficiency was assessed by observing GFP expression under a fluorescence microscope 24 h post-infection. For overexpression constructs, cells were selected with puromycin (Gibco, 1938660; dilution 1:10,000) to establish stably transduced lines. BMSCs were successfully transduced with lentiviruses carrying sh-NC, sh-PRDX6-1, sh-PRDX6-2, oe-NC, or oe-HIF-1α. qPCR was performed to validate the expression levels of target genes.

### 2.15 Animal experiments

Twenty 8-week-old male ICR mice (purchased from the Nantong University Experimental Animal Center) were anesthetized, and 15 μL of nanoparticle suspension was injected into the right knee joint cavity at fixed time points using a micro-injection syringe. To ensure uniform distribution of nanoparticles in the joint cavity, the suspension was sonicated in a carrier of mouse serum: phosphate-buffered saline (1:1) [31]. Based on the optimal concentration of SeNPs determined in *vitro*, and considering the range previously demonstrated to be safe and effective in alleviating oxidative stress in animal models [32], a dosage of 1 mg/kg was selected for subsequent experiments. The experimental groups were administered SeNPs (1 mg/kg), CoNPs (0.5 mg/kg) [33], and CoNPs (0.5 mg/kg) + SeNPs (1 mg/kg), while the control group received normal culture medium. Five mice were included in each group. After 8 w, the mice were euthanized, and blood was collected via cardiac puncture for analysis. The right knee joints and internal organs (heart, liver, spleen, lungs, and kidneys) were carefully dissected and fixed in neutral-buffered formalin. This study was conducted in accordance with all ethical guidelines for animal research, and the experimental protocol was approved by the Animal Ethics Committee of Nantong University (Approval No. S20250210-006).

### 2.16 Histology and immunofluorescence

After fixation, decalcification, dehydration, paraffin embedding, and sectioning (6 μm thick) of knee joint and organ specimens, the paraffin-embedded tissue sections were stained with hematoxylin and eosin (H&E) (Servicebio, G1076) and safranin O-fast green (Servicebio, C01053) according to the manufacturer's instructions. The Osteoarthritis Research Society International (OARSI) score was based on safranin O-fast green staining in each specimen, and cartilage cell number and thickness at the tibial plateau were quantified using ImageJ software. For immunofluorescence staining of knee joint paraffin sections, antigen retrieval was performed using citrate buffer, followed by permeabilization with 0.1 % Triton X-100 and blocking with 2 % BSA. Sections were incubated overnight with primary antibodies against GPX4 (Abcam, ab125066; dilution 1:200), HIF-1α (Abcam, ab308433; dilution 1:100), and HO-1 (Abcam, ab189491; dilution 1:250). After incubating with Alexa Fluor® 488 (Servicebio, GB25303; dilution 1:500) or Alexa Fluor® 594 (Servicebio, GB28301; dilution 1:500) goat anti-rabbit secondary antibodies, sections were stained with DAPI to label nuclei. Fluorescence images were captured using a fluorescence microscope, and the number or percentage of GPX4^+^ (green fluorescence), HIF-1α^+^ (red fluorescence), and HO-1^+^ (green fluorescence) cells in the target area was quantified using ImageJ software.

### 2.17 Statistical analysis

All datasets were organized and analyzed in Microsoft Excel 2019 and GraphPad Prism v.8.0.2 Software. All data presented are expressed as the mean ± s.d. The significance of the results was determined employing two-tailed unpaired Student's t-test (when comparing two groups) or one-way analysis of variance (ANOVA) with Tukey's correction for multiple comparisons (when more than two groups were compared) and significance is indicated in the related figure legends.

## 3 Results and discussion

### 3.1 Exploration of the *in vivo* molecular mechanisms underlying metallosis induced by cobalt-based metal implants

**Fig. 2:**
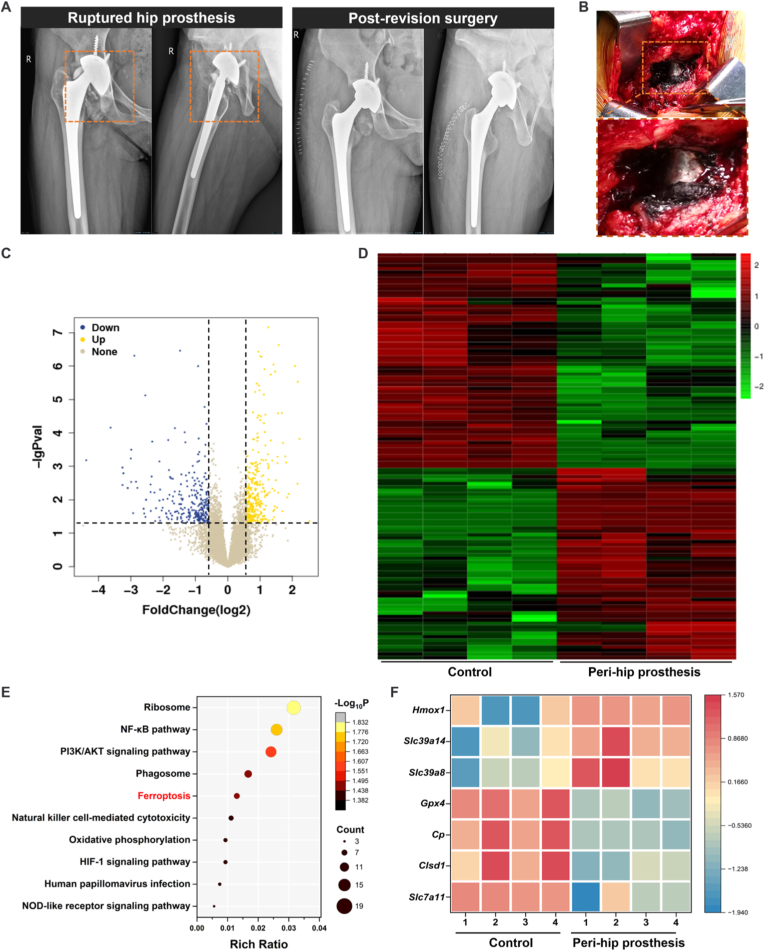
Total mRNA was extracted and subjected to RNA-sequence assay for gene expression identifying. (A) A patient with femoral head avascular necrosis underwent THA surgery. Over ten years post-surgery, the prosthetic femoral head fractured, dislocated, and required revision surgery. X-ray images of the right hip joint were taken both preoperatively and postoperatively. (B) During the second surgery, inflammatory pseudotumor and metal-related tissue darkening were observed around the artificial joint, presenting as symptoms of metallosis. (C) The volcano plot displays the differential regulation of gene expression between normal synovial tissue and synovial tissue surrounding cobalt-based metallic implants, as identified by RNA sequencing analysis. Genes upregulated and downregulated are shown in yellow and blue, respectively. (D) Hierarchically clustered heatmap of relative expression values for the most statistically significant genes. (E) Relevant signal pathways were evaluated by KEGG pathway analysis. (F) Heatmap of ferroptosis related genes identified in (E). (For interpretation of the references to color in this figure legend, the reader is referred to the Web version of this article.)

Orthopedic treatments for trauma, tumors, deformities, osteoporosis, and bone diseases often require the use of cobalt-containing metal implants [3]. Cobalt-containing metal prostheses are widely used as implant materials in THA (Fig. 2A). Due to the limited lifespan of these prostheses, some patients requiring revision surgeries exhibit pathological changes around the implant, such as tissue darkening and inflammatory pseudotumors (Fig. 2B). These manifestations are attributed to the accumulation of CoNPs to toxic concentrations, resulting in metallosis [33]. Previous studies have suggested that CoNPs induce macrophage infiltration, phagocytosis, and apoptosis, leading to clinical complications such as inflammation, osteolysis, and cytotoxicity [4,6]. The classic oxidative stress-related nuclear factor erythroid 2-related factor 2 (Nrf2) pathway is a key signaling pathway involved in the toxicological regulation of cobalt [10,34]. Apoptosis is considered the primary mechanism of cobalt-induced toxicity. However, the efficacy of various antioxidants in treating cobalt-induced apoptosis is generally limited, and high-confidence clinical sample reports are lacking [35].

To investigate the potential molecular mechanisms underlying the *in vivo* toxic response of cobalt-based alloys, we performed transcriptomic analysis on tissues surrounding the artificial joint in the hip joint cavity. RNA sequencing was conducted on four inflammatory synovial tissue samples collected during revision surgeries, with healthy synovial tissue samples from the initial hip replacement surgeries serving as controls. Cobalt-containing metal prostheses led to the upregulation of 399 genes and downregulation of 325 genes (Fig. 2C). A heatmap was used to comprehensively analyze the expression levels of differentially expressed genes (Fig. 2D). Due to the formation of inflammatory pseudotumors, KEGG enrichment analysis revealed significant enrichment in signaling pathways associated with inflammation and fibrosis, such as NF-κB and PI3K/AKT. Moreover, cobalt-containing metal prostheses also notably impacted the ferroptosis signaling pathway (Fig. 2E). Although inflammation is widely recognized as a hallmark response to metallic joint prostheses, our study specifically focused on ferroptosis. This decision was based on recent studies have shown that CoNPs induce dose-dependent neuronal cell death through a non-apoptotic mechanism, which involves lipid peroxidation, GSH depletion, and downregulation of GPX4, suggesting that CoNPs may contribute to neurodegenerative damage by inducing ferroptosis [36]. Our previous research also demonstrated that CoNPs reduce the viability of mouse fibroblast cell line BALB/3T3 in a manner similar to ferroptosis [15]. The ferroptosis pathway was selected as the mechanistic focus due to its novelty, distinct regulatory features, and strong experimental support. Further analysis of the clustering heatmap of ferroptosis-related genes revealed their involvement in regulating metabolism, oxidative stress response, autophagy, and iron ion transport, all of which are crucial for ferroptosis (Fig. 2F) [13]. Sequencing results of human samples confirmed that ferroptosis is closely associated with the metallosis induced by cobalt-containing metallic prostheses *in vivo*.

### 3.2 Synthesis and characterization of SeNPs

Currently, chelation therapy is the predominant clinical approach for detoxification of CoNPs [37]. However, the continuous release of metal ions and particles within the joint cavity remains challenging to fully chelate. In some cases, plasma exchange or even revision surgery to replace the implant may be required [35]. These passive detoxification methods, especially multiple revision surgeries, cause significant discomfort for patients, highlighting the urgent need for effective new detoxification strategies and approaches in clinical practice.

**Fig. 3:**
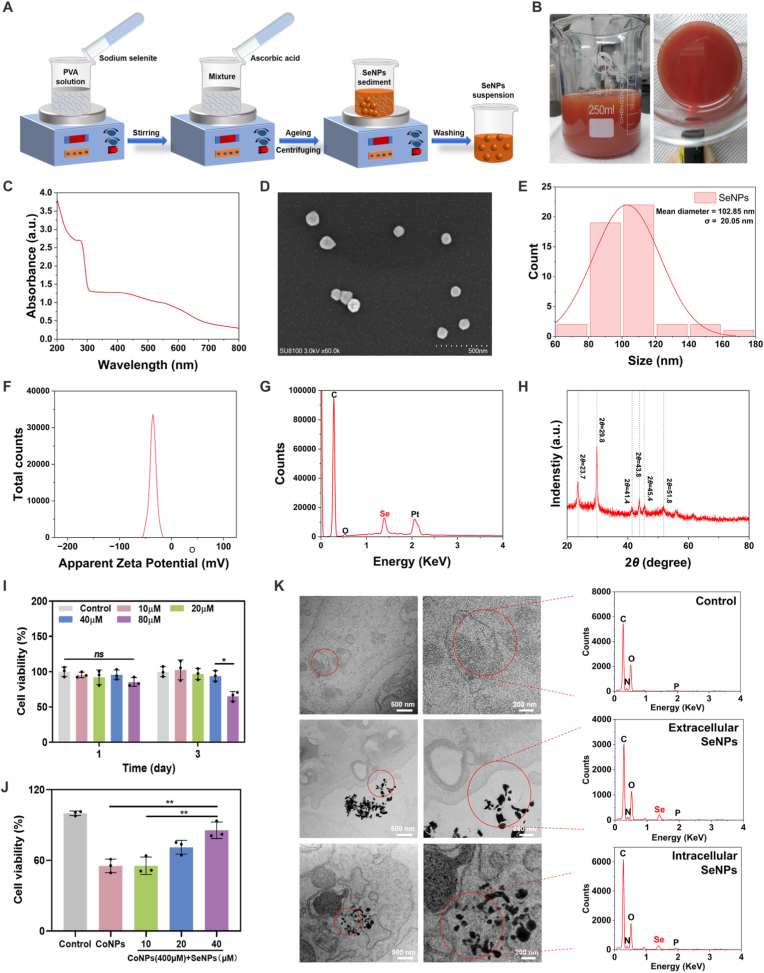
Preparation and characterization of SeNPs. (A) Schematic diagram illustrating the chemical reduction method for the preparation of SeNPs. (B) SeNPs were uniformly dispersed in a brick-red suspension. (C) UV–vis absorption spectra of SeNPs at 48 h. (D) SEM image of SeNPs. Scale bar = 500 nm. (E) Particle size distribution of SeNPs. (F) Zeta potential of SeNPs. (G) EDX analysis of SeNPs in point scanning mode. (H) XRD pattern of SeNPs. (I) CCK-8 assay results for BMSCs cultured in medium containing different concentrations of SeNPs for 1 day and 3 days (n = 3). (J) CCK-8 assay results for BMSCs co-cultured with 400 μM CoNPs and different concentrations of SeNPs for 1 day (n = 3). (K) TEM-EDX images of BMSCs co-incubated with SeNPs for 24 h. The *p*-values by one-way ANOVA are indicated. *ns*: no significance, *∗p* < 0.05, *∗∗p* < 0.01. (For interpretation of the references to color in this figure legend, the reader is referred to the Web version of this article.)

Guided by the RNA sequencing data, SeNPs with anti-ferroptotic bioactivity hold promise for targeted active detoxification directly at the source of cobalt-based metallic implants. SeNPs are chemically synthesized using sodium selenite, with ascorbic acid as a reducing agent and PVA as a stabilizer, as shown in Fig. 3A. Compared to other reducing agents, ascorbic acid is typically the preferred choice due to its good biocompatibility and low *in vivo* toxicity [38]. During the preparation of SeNPs, the transition of the colorless solution to a "brick-red" hue indicates successful formation of SeNPs (Fig. 3B), which is associated with surface plasmon resonance phenomena [39,40]. The formation and surface plasmon vibration of SeNPs were characterized by UV–Vis spectroscopy, displaying a broad peak at 270 nm (Fig. 3C). Fig. 3D shows the SEM image of SeNPs, which demonstrates good dispersion and a spherical morphology. The average particle size of the nanoparticles was found to be 102.85 ± 20.05 nm (Fig. 3E), which is favorable for cellular uptake [41]. Moreover, the particle size remained largely unchanged after 4 weeks of storage at room temperature (Fig. S1), indicating that the SeNPs possess excellent long-term colloidal stability. The zeta potential of SeNPs was −34.78 mV (Fig. 3F), indicating good dispersion stability in aqueous solutions. The elemental composition of the nanoparticles was analyzed using EDS (Fig. 3G). Absorption peaks were observed at 1.20, 1.24, and 1.38 keV, corresponding to specific selenium absorption peaks [42]. In addition, signals for C, O, Al, and Pt were detected, which are associated with the grid substrate and conductive coating used in the SEM-EDS analysis (Fig. S2). XRD analysis of the nanoparticles revealed distinct and stable Bragg reflections (Fig. 3H). The diffraction peaks at 2*θ* values of 23.7, 29.8, 41.4, 43.8, 45.4, and 51.8° confirmed the formation of SeNPs [39], demonstrating that the nanoparticles synthesized via chemical reduction are indeed SeNPs.

BMSCs are pluripotent mesenchymal stem cells capable of differentiating into various cell phenotypes surrounding the joint, including osteoblasts, chondrocytes, and adipocytes. This makes them an ideal cell type for evaluating cobalt toxicity and detoxification efficacy within the joint cavity. The size of the wear and corrosion particles extracted from tissues surrounding the implant is known to be approximately 30∼40 nm [43], which is comparable to the size of the CoNPs used in this study. In our previous study, the half-maximal inhibitory concentration (IC_50_) of CoNPs was determined to be approximately 400 μM [15], a value that lies within the reported range of CoNPs accumulation in clinical patients [44]. However, we were unable to directly measure cobalt concentrations in periprosthetic synovial tissues due to the limited availability of patient samples, and such levels may vary considerably depending on implantation time, patient activity, and prosthesis positioning. Consistently, co-incubation of BMSCs with 400 μM CoNPs markedly reduced cell viability (Fig. S3). However, after CoNPs exposure, only a few scattered TUNEL-positive apoptotic cells were observed (Fig. S4), suggesting that traditional apoptosis is not the primary mechanism underlying the decreased viability of BMSCs induced by CoNPs. This indirectly supports the idea that ferroptosis, a novel non-apoptotic form of cell death, plays an important role in the cytotoxicity of CoNPs. Next, we added different concentrations of SeNPs to the culture medium to investigate their impact on BMSC viability. In short-term co-cultures, no significant differences in cell viability were observed among the groups. However, after three days, a significant decrease in cell viability was observed in the 80 μM SeNPs group (Fig. 3I). Live/Dead staining also revealed a large number of dead cells only in the 80 μM SeNPs group (Fig. S5). These results collectively indicate that lower concentrations of SeNPs (≤40 μM) exhibit good biocompatibility with BMSCs. Subsequently, we treated BMSCs with 400 μM CoNPs and varying concentrations of SeNPs for 24 h. The highest cell viability was observed in the 40 μM SeNPs group (Fig. 3J), thus determining 40 μM as the optimal detoxification concentration of SeNPs for subsequent cell experiments. Finally, TEM was employed to directly examine whether SeNPs could be efficiently internalized by BMSCs. Following 24 h of incubation with 40 μM SeNPs, TEM images revealed distinct electron-dense black dots within the cytoplasm (Fig. 3K). Subsequent EDX point scanning of these regions confirmed the presence of selenium-specific signals, demonstrating that SeNPs were effectively internalized by BMSCs (Fig. 3K).

### 3.3 SeNPs suppresses CoNPs-induced ferroptosis in BMSCs

**Fig. 4:**
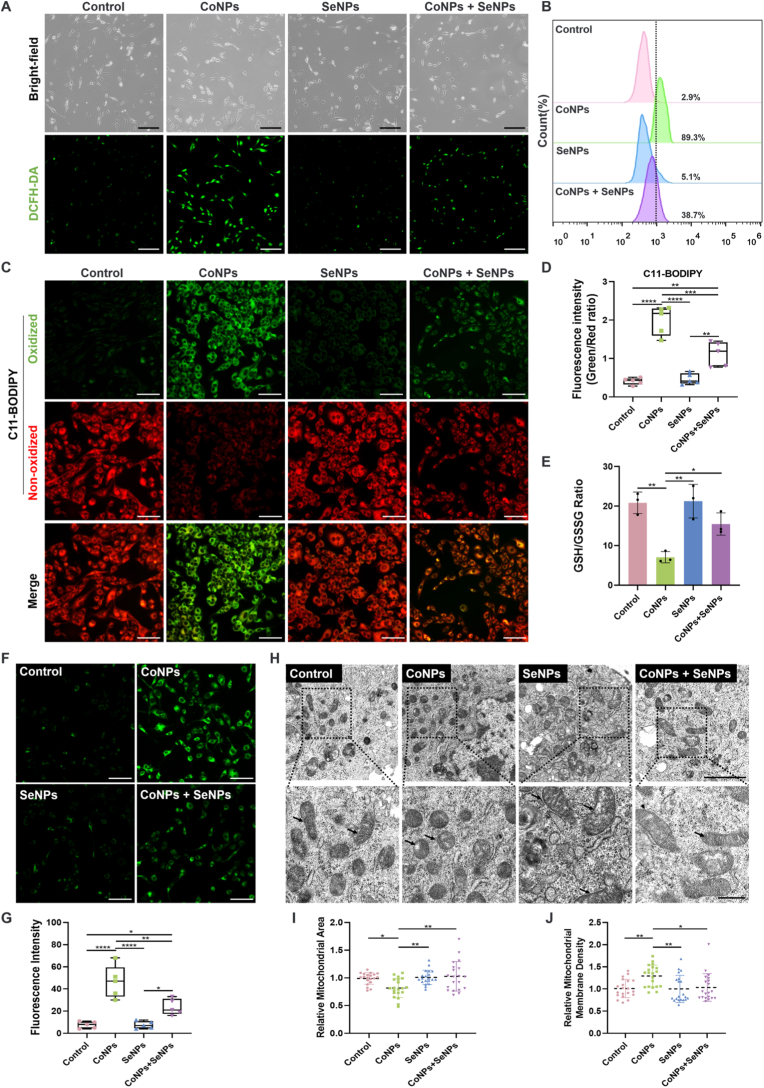
SeNPs suppresses CoNPs-induced ferroptosis in BMSCs. (A) Representative fluorescence (DCFH-DA staining) and corresponding brightfield images of ROS staining in BMSCs following treatment with various nanoparticles. Scale bar = 200 μm. (B) Flow cytometry analysis of FITC-ROS in each group. (C) Fluorescence images assessing lipid peroxidation in BMSCs treated with different nanoparticles using C11-BODIPY. Scale bar = 100 μm. (D) Green fluorescence intensity divided by red fluorescence intensity was used to determine the relative level of lipid peroxidation (n = 5). (E) GSH/GSSG in BMSCs were quantitatively determined using an GSSG/GSH Quantification Kit (n = 3). (F) Representative images of Mito-FerroGreen staining in BMSCs. Scale bar = 100 μm, and (G) quantification of Fe^2+^ levels (n = 5). (H) Bio-TEM images of the mitochondrial morphology in BMSCs after incubation with different nanoparticles; scale bar = 2 μm, 500 nm, respectively. (I) Relative area and (J) density of mitochondrial was quantitative analysis by using the ImageJ software (n = 20). The *p*-values by one-way ANOVA are indicated. *∗p* < 0.05, *∗∗p* < 0.01, *∗∗∗p* < 0.001, *∗∗∗∗p* < 0.0001. (For interpretation of the references to color in this figure legend, the reader is referred to the Web version of this article.)

To verify the ferroptosis-targeted detoxification effect of SeNPs, we focused on investigating the impact of CoNPs and SeNPs on the biochemical processes of ferroptosis. Functional experiments on CoNPs-induced oxidative stress and SeNPs-mediated antioxidant activity were first conducted using the DCFH-DA probe. CoNPs induced the accumulation of intracellular ROS, as evidenced by an increase in DCF green fluorescence intensity. Upon the introduction of SeNPs, ROS production in BMSCs was significantly reduced (Fig. 4A). The quantitative analysis of ROS levels by flow cytometry was consistent with the fluorescence imaging results (Fig. 4B and Fig. S6), collectively demonstrating that SeNPs have the ability to scavenge CoNPs-induced ROS. Ferroptosis is a form of cell death induced by an increase in intracellular iron accumulation, which leads to elevated ROS levels and lipid peroxidation [13]. The effect of CoNPs and SeNPs on lipid peroxidation was further assessed (Fig. 4C). Compared to stimulation with CoNPs alone, the introduction of SeNPs partially reversed the increase in lipid ROS levels induced by CoNPs treatment (Fig. 4D). Cellular lipid peroxidation is a key feature of ferroptosis and exhibits a similar trend to that of ROS levels. These findings further validated the ROS toxicity of CoNPs and the antioxidant effect of SeNPs. GPX4 detoxifies lipid hydroperoxides into non-toxic lipid alcohols using GSH, thereby inhibiting ferroptosis [45,46]. The depletion of GSH leads to GPX4 inactivation, inducing lipid peroxidation and ferroptosis. Therefore, the effect of CoNPs and SeNPs on intracellular GSH levels was subsequently assessed using the GSSG/GSH quantitative assay kit. CoNPs significantly reduced the GSH/GSSG ratio, whereas the co-exposure group (CoNPs + SeNPs) markedly increased the GSH/GSSG ratio (Fig. 4E). These results suggest that CoNPs deplete GSH and reduce the cellular antioxidant capacity, whereas SeNPs help maintain cellular antioxidant function. Fe^2+^ generates hydroxyl radicals through the Fenton reaction, further inducing lipid peroxidation and leading to ferroptosis [13]. Further investigation is needed to evaluate the effect of CoNPs and SeNPs on intracellular Fe^2+^ concentration. The fluorescence intensity of Mito-FerroGreen (Fig. 4F), a specific probe for detecting Fe^2+^ within mitochondria, significantly increased after CoNPs treatment, indicating that CoNPs led to a substantial increase in intracellular Fe^2+^ concentration, disrupting iron metabolism and activating the ferroptosis pathway. However, this increase in iron levels was partially reversed by the addition of SeNPs (Fig. 4G). Given the association between ferroptosis and mitochondrial dysfunction, we observed the mitochondrial morphology in each group using TEM. In BMSCs exposed to CoNPs for 24 h, mitochondrial morphological changes characteristic of ferroptosis were observed (Fig. 4H), including reduced mitochondrial size, increased membrane density, and the loss or reduction of cristae (Fig. 4I and J). However, the addition of SeNPs rescued the CoNPs-induced mitochondrial morphological changes, suggesting that CoNPs induce ferroptosis in BMSCs, and SeNPs function as an inhibitor. In addition to the ultrastructural features observed by TEM, we further performed a functional assay measuring ATP production. Consistent with the morphological changes, CoNPs exposure markedly reduced ATP levels, whereas SeNPs treatment partially restored ATP production (Fig. S7), providing complementary functional evidence of mitochondrial impairment during ferroptosis. Future studies will incorporate the assessment of mitochondrial membrane potential to achieve a more comprehensive characterization of mitochondrial dysfunction in CoNPs-induced ferroptosis.

Collectively, the results from these experiments demonstrate that CoNPs induce the accumulation of total ROS, lipid peroxidation, and a decrease in intracellular GSH levels, leading to increased iron accumulation and ferroptosis in BMSCs. However, these toxic effects can be partially reversed by SeNPs.

### 3.4 SLC7A11/GPX4 and HIF-1α/HO-1 mediate SeNPs detoxifying cobaltism via inhibiting ferroptosis

**Fig. 5:**
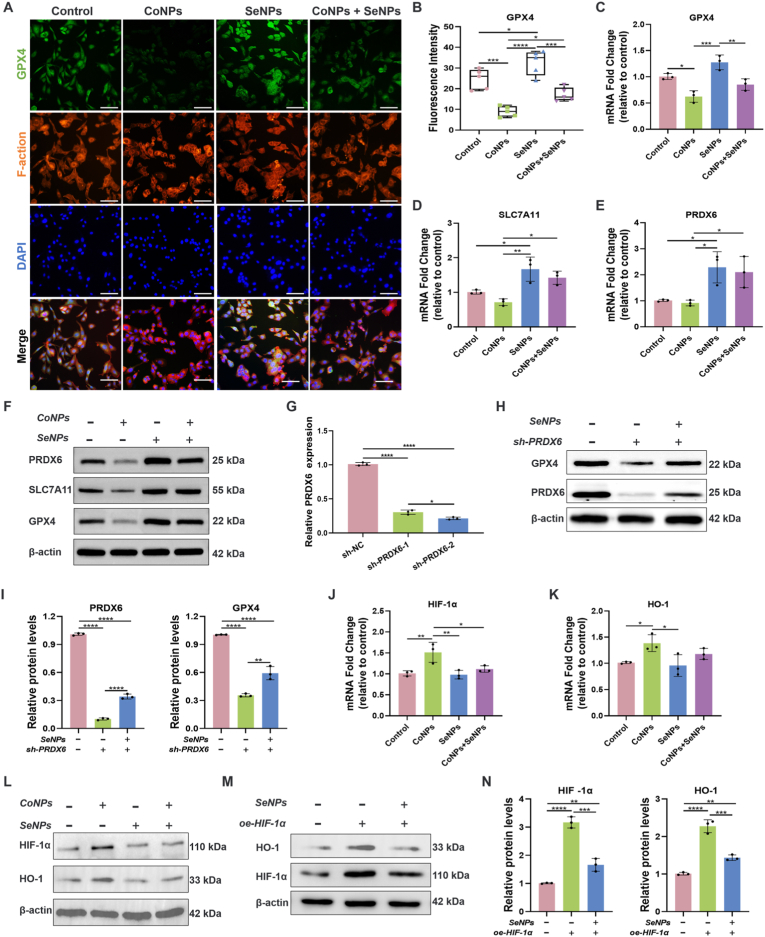
SLC7A11/GPX4 and HIF-1α/HO-1 mediate SeNPs detoxifying cobaltism via inhibiting ferroptosis. (A) Immunofluorescence staining of GPX4 in BMSCs treated with different nanoparticles. Scale bar = 100 μm. (B) Semiquantitative analysis of the fluorescence intensity of GPX4 (n = 5). (C–E) qRT-PCR analysis of ferroptosis suppressor genes (*PRDX6*, *GPX4*, and *SLC7A11*) in BMSCs treated with different nanoparticles for 48 h. (F) Western blot analysis of PRDX6, SLC7A11, and GPX4 protein expression in BMSCs treated with different nanoparticles. (G) qRT-PCR analysis of the knockdown efficiency of shPRDX6-1 and shPRDX6-2 in BMSCs. (H) Representative Western blot showing GPX4 expression in BMSCs following PRDX6 knockdown and treatment with 40 μM SeNPs. (I) Relative protein levels of PRDX6 and GPX4 were assessed by Western blotting following PRDX6 knockdown using shRNA. qRT-PCR analysis of *HIF-1α* (J) and *HO-1* (K) genes in BMSCs treated with different nanoparticles for 48 h. (L) Western blot analysis of HIF-1α and HO-1 protein expression in BMSCs treated with different nanoparticles. (M) Representative Western blot showing HO-1 expression in BMSCs treated with SeNPs (40 μM, 24 h) following HIF-1α overexpression. (N) Western blot analysis of HIF-1α and HO-1 protein levels in BMSCs following HIF-1α overexpression. The *p*-values by one-way ANOVA are indicated. *∗p* < 0.05, *∗∗p* < 0.01, *∗∗∗p* < 0.001, *∗∗∗∗p* < 0.0001.

GPX4 is the only peroxidase in mammals capable of reducing phospholipid hydroperoxides in cellular membranes [45]. It plays a critical role in maintaining lipid homeostasis and preventing the accumulation of toxic lipid ROS [46]. To investigate the mechanism by which SeNPs reverse CoNPs-induced ferroptosis, we first assessed the level of GPX4 through immunofluorescence staining (Fig. 5A). The green fluorescence intensity in the SeNPs group was approximately 1.4 times higher than in the normal control group. However, after CoNPs stimulation, the fluorescence intensity of GPX4 significantly decreased. When BMSCs were co-incubated with both types of nanoparticles, the fluorescence intensity of GPX4 was restored to near normal levels (Fig. 5B). These results indicate that GPX4 plays a key role in the SeNPs-mediated attenuation of CoNPs-induced ferroptosis-related cytotoxicity.

The Solute carrier family 7 member 11 (SLC7A11)/GPX4 axis is one of the most critical defense pathways against ferroptosis. Further investigation revealed changes in the expression levels of the *GPX4* and *SLC7A11* genes (Fig. 5C and D). Consistent with the immunofluorescence staining of GPX4, CoNPs treatment resulted in downregulation of both *GPX4* and *SLC7A11* gene expression. In contrast, SeNPs exhibited the opposite trend, with a significant increase in the expression levels of both *GPX4* and *SLC7A11* genes. Moreover, co-exposure to CoNPs and SeNPs rescued the downregulation of ferroptosis markers *GPX4* and *SLC7A11* induced by CoNPs. This phenomenon can be attributed to the typical biological function of selenium, which is to generate selenocysteine (Sec) residues in selenoproteins, forming the basis for its role as an essential antioxidant and cellular protective micronutrient [16]. Peroxiredoxin 6 (PRDX6) is a novel selenium protein synthesis factor that functions as a selenium delivery protein to enhance the utilization efficiency of selenium [47]. It facilitates the efficient synthesis of Sec-tRNA, thereby accelerating the translation of selenium-dependent proteins such as GPX4 [48]. This process helps to combat ferroptosis without disrupting iron metabolism [49]. Further research is required to determine the role of PRDX6 in SeNPs-mediated inhibition of BMSC ferroptosis and whether the transcriptional regulation of PRDX6 contributes to CoNPs-induced ferroptosis. qRT-PCR results indicated that CoNPs treatment alone did not affect the expression of PRDX6, whereas BMSCs exposed to SeNPs for 48 h showed high expression of *PRDX6* (Fig. 5E). Western blotting analysis revealed a similar, though more pronounced, trend (Fig. 5F, and Fig. S8). SeNPs completely reversed the downregulation of ferroptosis inhibitory proteins (GPX4 and SLC7A11) induced by CoNPs and significantly upregulated their expression. SeNPs also promoted the protein expression of PRDX6, which in turn enhanced GPX4 expression. GPX4 detoxifies lipid hydroperoxides by catalyzing Sec residues, thereby inhibiting ferroptosis [49]. Therefore, PRDX6 acts as an independent factor and plays a positive role in SeNPs-mediated prevention of CoNPs-induced lipid peroxidation and ferroptosis. To further investigate the regulatory relationship between PRDX6 and GPX4 following SeNPs intervention, we designed two shRNA sequences targeting PRDX6 and selected sh-PRDX6-2, which exhibited higher knockdown efficiency, for subsequent experiments (Fig. 5G). Western blot analysis revealed that knockdown of PRDX6 in BMSCs led to a reduction in GPX4 expression (Fig. 5H). Notably, treatment with SeNPs partially reversed this effect, as evidenced by a relative increase in PRDX6 and a marked upregulation of GPX4 (Fig. 5I). These findings suggest that SeNPs may promote GPX4 expression by upregulating PRDX6. In future studies, overexpression and co-immunoprecipitation (Co-IP) experiments targeting PRDX6 will be necessary to provide more direct evidence for its interaction with GPX4 and other key regulators of ferroptosis.

Cobalt can act as a hypoxia mimetic in cell cultures, directly inhibiting the activity of prolyl hydroxylases that promote the degradation of Hypoxia-inducible factor-1 alpha (HIF-1α), leading to its accumulation at the protein level [50]. The HIF-1α pathway plays a crucial role in cobalt-induced cytotoxicity, being involved in iron homeostasis and lipid peroxidation, and is a key driver of the targeting of GPX4 [51]. Our findings support this mechanism, as CoNPs exposure activated the HIF-1α pathway, with significant increases observed in both *HIF-1α* gene (Fig. 5J)and protein levels (Fig. 5L). HIF-1α translocates to the nucleus and binds to hypoxia response elements (HREs), initiating the transcription of downstream genes [52]. Among these, heme oxygenase-1 (HO-1) is a critical downstream molecule of the HIF-1α signaling pathway [53]. HO-1 catalyzes the degradation of heme, producing carbon monoxide, biliverdin, and free iron, and has antioxidant, anti-inflammatory, and cytoprotective properties [53,54]. However, accumulating evidence suggests that HO-1 is a key mediator in the progression of ferroptosis [55]. Overexpression of HO-1 promotes cellular oxidative stress and alterations in mitochondrial permeability, facilitating the influx of iron into the mitochondrial matrix, ultimately leading to mitochondrial cristae fragmentation, degeneration, and ferroptosis [55,56]. Clearly, HO-1 plays a dual role in maintaining iron and ROS homeostasis, as well as in the induction of ferroptosis. Two alternative splicing isoforms of HO-1 have been identified, with sizes of 750 bp and 250 bp [57], which may help elucidate the multifunctional role of HO-1 in different cellular processes. Previous studies have shown that cobalt exposure increases cellular ROS levels and, through activation of the Keap1-Nrf2-ARE antioxidant signaling pathway, promotes HO-1 expression [58]. We propose that the increase in *HO-1* following CoNPs exposure is also due to the activation of the HIF-1α/HO-1 pathway (Fig. 5K and L). In this study, the expression of HIF-1α and HO-1 was restored to near normal levels upon the addition of SeNPs (Fig. S8). However, SeNPs alone did not alter the HIF-1α/HO-1 signaling pathway, further suggesting that SeNPs modulate the HIF-1α/HO-1 pathway specifically under the condition of CoNPs-induced ferroptosis. To further investigate whether SeNPs inhibit ferroptosis by modulating the HIF-1α/HO-1 signaling pathway, we generated BMSCs with HIF-1α overexpression. Western blot analysis showed that infection with oe-HIF-1α significantly activated the HIF-1α/HO-1 signaling cascade, as evidenced by increased HO-1 expression (Fig. 5M). Upon SeNPs treatment, this activation was partially reversed, with a noticeable reduction in HIF-1α levels, leading to a subsequent downregulation of HO-1(Fig. 5N). These results suggest that CoNPs induce ferroptosis via activation of the HIF-1α/HO-1 signaling pathway, while SeNPs suppress ferroptosis by downregulating HIF-1α and consequently reducing HO-1 expression.

These results reveal that CoNPs induce ferroptosis through the inhibition of the SLC7A11/GPX4 axis and activation of the HIF-1α/HO-1 signaling pathway. SeNPs promote high expression of PRDX6 in cells, enhancing the activity of the selenoprotein GPX4 and inhibiting the activation of the HIF-1α/HO-1 signaling pathway, thereby detoxifying the ferroptosis pathway. In this study, we employed multipotent BMSCs as a representative cell type to evaluate the effects of CoNPs and SeNPs on ferroptosis-related signaling pathways. In future investigations, it will be necessary to explore the distinct mechanisms by which these nanoparticles affect other complex cell populations surrounding joint prostheses, including osteoblasts, chondrocytes, and synoviocytes.

### 3.5 SeNPs ameliorates CoNPs-induced synovial tissues and cartilage tissues ferroptosis in mouse knee joints

**Fig. 6:**
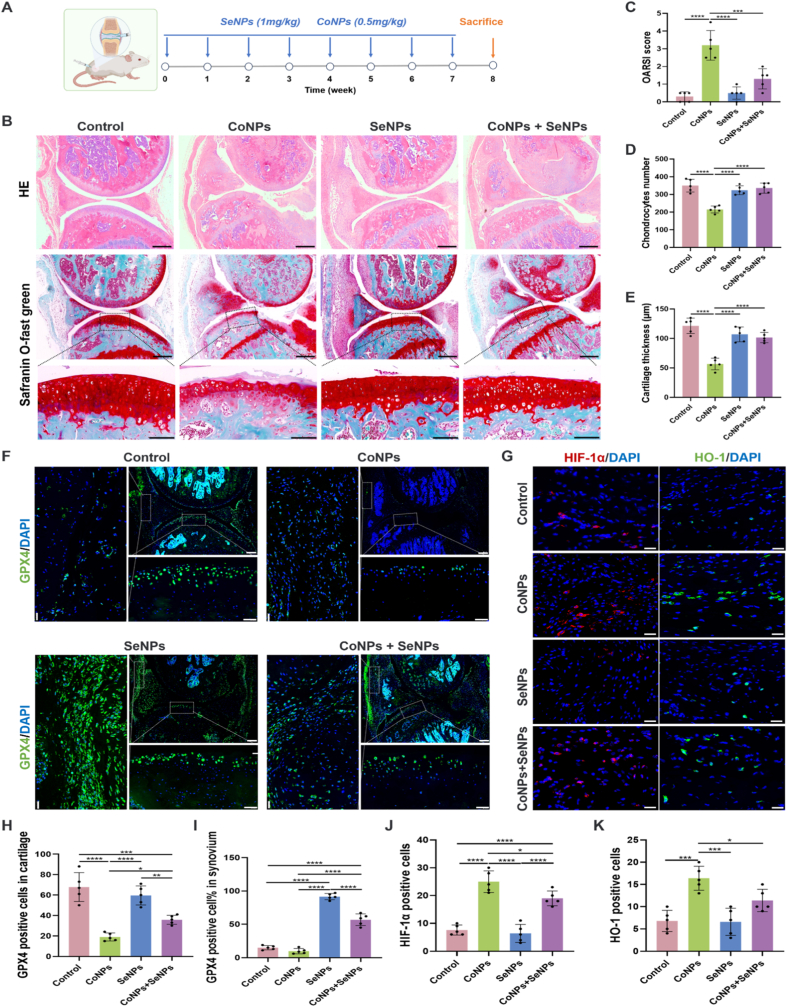
SeNPs ameliorates CoNPs-induced synovial tissues and cartilage tissues ferroptosis in mouse knee joints. (A) Schematic diagram of the animal experimental procedure. (B) HE staining and Safranin-O/fast green staining were performed to observe the histopathological changes in the mouse knee joints following nanoparticle injection. Scale bar = 200 μm, 200 μm, 50 μm, respectively. (C) OARSI grade used for evaluation of the cartilage degradation in the four groups. Quantitation of chondrocytes numbers (D) and cartilage thickness (E). (F) Representative images for GPX4 immunostaining in synovial tissues (left) and cartilage tissues (down) obtained from different group mouse knees. Scale bar = 20 μm, 200 μm, 40 μm, respectively. (G) Representative images for HIF-1α (left) and HO-1 (right) immunostaining in synovial tissues obtained from different group mouse knees. Scale bar = 20 μm. (H) The bar graphs show quantification of the GPX4-positive cells from total cell population per field, in cartilage immunofluorescence sections. (I) The proportion of GPX4-positive cells in the synovial tissue. (J) The bar graphs show quantification of the HIF-1α or (K) HO-1-positive cells from total cell population per field, in synovial tissues immunofluorescence sections. n = 5 per group; The *p*-values by one-way ANOVA are indicated. *∗p* < 0.05, *∗∗p* < 0.01, *∗∗∗p* < 0.001, *∗∗∗∗p* < 0.0001. (For interpretation of the references to color in this figure legend, the reader is referred to the Web version of this article.)

In the *in vivo* experiments, due to the small volume of the mouse hip joint, it was difficult to achieve accurate injection. Additionally, the volume of nanoparticle suspension that could be accommodated while maintaining the integrity of the joint capsule was limited. Therefore, we selected the knee joint, which offers more space, for precise injection of an adequate dose of nanoparticles (Fig. 6A). However, this model also has certain limitations. The nanoparticle delivery was performed through multiple injections rather than continuous infusion or slow release, which does not replicate the clinical scenario of CoNPs being continuously and stably released from joint prostheses. After weekly injections for 8 weeks, the biochemical indicators in the peripheral blood of the mice remained within normal ranges (Fig. S9), and no significant toxic reactions were observed in the major internal organs (heart, liver, spleen, lung, and kidney) (Fig. S10). Furthermore, no pathological changes such as fibrosis, inflammation, or necrosis were detected, which is consistent with previous reports [31,33]. This suggests that in this model, nanoparticles injected at specific intervals did not cause off-target effects. A portion of the nanoparticles accumulated in the joint cavity, while the remainder was rapidly cleared from the systemic circulation. In contrast, the elevated peripheral blood Co^2+^ levels and organ cobalt accumulation observed clinically in patients after MOM-THA result from the continued generation of CoNPs and Co^2+^ in the body [4,59].

Hematoxylin and eosin (HE) staining of sagittal sections of the mouse knee joint showed that (Fig. 6B), compared to the control group, the synovial membrane in the three nanoparticle injection groups exhibited thickening of the villous walls. This was likely related to repeated nanoparticle injections into the joint cavity and the local stimulation caused by the nanoparticles. Safranin-O/fast green staining (Fig. 6B) revealed that the sole injection of SeNPs did not alter the OARSI score (Fig. 6C), nor did it affect the number of chondrocytes (Fig. 6D) or the cartilage thickness (Fig. 6E). In the CoNPs injection group, severe proliferation of the synovial tissue around the knee joint, wear on the knee joint surface, and cartilage degeneration and destruction were observed, along with signs of joint deformity, including narrowing of the joint space and osteophyte formation (Fig. 6B). Quantitative analysis showed a significant increase in the OARSI score, a reduction in the number of chondrocytes, and a marked decrease in cartilage thickness in the tibial joint surface. However, the concurrent injection of SeNPs significantly alleviated cartilage degeneration, reduced the OARSI score, and exerted a protective effect against CoNPs-induced joint toxicity (Fig. 6C–E). Immunofluorescence staining revealed a significant reduction in GPX4 expression in chondrocytes in the CoNPs injection group (Fig. 6F), which was partially reversed when SeNPs and CoNPs were co-injected (Fig. 6H). A similar and even more pronounced trend was observed in the synovial tissue, where the downregulation of GPX4 expression induced by CoNPs was rescued by the co-injection of SeNPs (Fig. 6F). Specifically, the proportion of GPX4-positive synovial cells in the SeNPs and SeNPs + CoNPs groups was significantly higher than in the normal control group (Fig. 6I). Additionally, synovial cell proliferation was notably increased and densely packed in CoNPs injection group, which was consistent with the thickening of the synovium observed in the HE staining. Further investigations revealed that CoNPs exacerbated hypoxia in the synovial tissue and led to a significant increase in the immunofluorescence intensity of HIF-1α and its downstream key molecule HO-1 (Fig. 6G). In contrast, SeNPs significantly improved synovial hypoxia and downregulated the expression of HIF-1α and HO-1 (Fig. 6J and K).

These results are consistent with the *in vitro* findings and collectively suggest that CoNPs may promote ferroptosis, leading to toxicity manifested as synovial tissue hyperplasia, wear on the knee joint surface, and cartilage degeneration and destruction. Synovial tissue proliferation is widely recognized to be closely associated with inflammation. Recent studies have revealed a mechanistic interplay between inflammation, ferroptosis, and synovial hyperplasia. In both rheumatoid arthritis patients and animal models, activation of the PI3K/AKT/mTOR inflammatory signaling pathway suppresses GPX4 expression, thereby promoting ferroptosis. This process contributes to the abnormal activation of synovial fibroblasts, ultimately leading to synovial hyperplasia and sustained inflammation [60,61]. Similarly, in hyperplastic synovial tissues from patients with osteoarthritis, reduced levels of GPX4 and GSH, along with elevated intracellular Fe^2+^, have been reported [62]. Consistent with these findings, our KEGG enrichment analysis of periprosthetic synovial tissues demonstrated marked activation of inflammatory pathways—including NF-κB and PI3K/AKT—as well as ferroptosis signaling (Fig. 2E). These results suggest a potential mechanistic link between inflammation and ferroptosis in synovial pathology. Further investigations are required to delineate the precise molecular crosstalk underlying CoNPs-induced synovial proliferation. In contrast, SeNPs effectively inhibit CoNPs-induced ferroptosis by actively upregulating GPX4 expression and downregulating the HIF-1α/HO-1 signaling pathway, thereby counteracting the series of pathological changes induced by cobalt toxicity within the knee joint cavity. A limitation of this study is the relatively small sample size used in the in *vivo* experiments (n = 5 per group). Nevertheless, the trends observed remain consistent and biologically meaningful. Future studies with larger cohorts are warranted to validate these results and further strengthen the translational relevance of our conclusions.

In summary, this study identifies ferroptosis as a key mechanism underlying CoNPs-induced joint toxicity and demonstrates that SeNPs can effectively mitigate this damage by suppressing ferroptosis, thereby protecting joint-relevant cells. Our findings support the potential of SeNPs as a novel surface modification or coating strategy to enhance the biocompatibility and long-term safety of cobalt-containing metal prostheses. Although our current animal model does not fully mimic the gradual and chronic release of CoNPs observed in clinical settings, it nevertheless provides important *in vivo* evidence and a theoretical foundation for targeted detoxification strategies. Looking ahead, future studies may focus on the development of selenium-based prostheses incorporating controlled-release properties, which could achieve sustained detoxification and more closely reflect clinical scenarios. Such approaches not only hold promise for improving the safety of orthopedic implants but also offer a feasible pathway toward clinical translation. However, our study lacks a comprehensive evaluation of the *in vivo* pharmacokinetics, dose-optimization and biodistribution of SeNPs, which remains a critical area for future investigation to better assess their behavior and long-term safety *in vivo*. Another limitation of this study is the lack of direct measurement of cobalt levels in periprosthetic synovial tissue. Future studies involving larger patient cohorts and direct quantification of cobalt in periprosthetic synovial tissue will be essential to further validate the clinical translational significance of our findings.

## 4 Conclusion

In this experiment, we demonstrated that CoNPs induce cellular ferroptosis and toxicity via the SLC7A11/GPX4/HIF-1α/HO-1 signaling pathway, while SeNPs detoxify by upregulating PRDX6, inhibiting the ferroptosis pathway. This study explores the non-apoptotic toxicity mechanism of CoNPs generated from wear of joint prostheses, focusing on the perspective of ferroptosis. It reveals a novel mechanism in which SeNPs inhibit ferroptosis to actively and targeted mitigate cobalt toxicity at its source. This finding provides important guidance for the development of selenium-coated artificial joint prostheses.

## CRediT authorship contribution statement

**Pengcheng Xu:** Writing – original draft, Software, Resources, Methodology, Formal analysis, Data curation, Conceptualization. **Fan Liu:** Writing – original draft, Resources, Project administration, Funding acquisition, Data curation. **Su Jiang:** Software, Project administration, Methodology, Data curation. **Baisheng Cai:** Resources, Methodology, Investigation, Data curation. **Cong Ye:** Software, Project administration, Methodology. **Yiming Sun:** Methodology, Data curation. **Yaping Wang:** Resources, Methodology. **Jining Shen:** Visualization, Validation. **Huan Zhou:** Writing – review & editing, Validation, Software, Methodology, Funding acquisition. **Yake Liu:** Writing – review & editing, Software, Resources, Methodology, Funding acquisition, Conceptualization.

## Declaration of competing interest

The authors declare that they have no known competing financial interests or personal relationships that could have appeared to influence the work reported in this paper.

## Data Availability

Data will be made available on request.
