# Supplementary material for: Selenium nanoparticles alleviate cobalt toxicity in artificial joint metal prostheses by inhibiting ferroptosis through activation of the PRDX6/GPX4 pathway
Source: Mater Today Bio. 2025 Sep 12;35:102306. doi: 10.1016/j.mtbio.2025.102306 (PMC12465050; doi:10.1016/j.mtbio.2025.102306)
Supplement: Multimedia component 1 [file mmc1.docx]

Supplementary materials for

**Selenium nanoparticles alleviate cobalt toxicity in artificial joint metal prostheses by inhibiting ferroptosis through activation of the PRDX6/GPX4 pathway**

1. **Materials and Methods**

**1.1 Terminal deoxynucleotidyl transferase dUTP Nick End Labeling (TUNEL) staining**

BMSCs were cultured in medium containing 400 µM CoNPs for 24 hours. As a control, cells cultured under normal conditions were used. Apoptosis in both groups was assessed using a TUNEL apoptosis detection kit (Vazyme, A111-01), and images were captured using an inverted fluorescence microscope.

**1.2 Live/Dead cell staining**

Different concentrations of SeNPs (range from 10 to 80 μM) were added to the culture medium, and BMSCs were cultured for 3 days. The culture medium was then replaced with Live/Dead fluorescent staining solution, and after incubation in the dark, images were captured using a fluorescence microscope.

**1.3. ATP production**

Cellular ATP levels were quantified using an ATP Assay Kit (Beyotime, S0026B ). After 24 h of co-incubation of BMSCs with the indicated nanoparticles, cells were harvested and lysed in ice-cold lysis buffer. The lysates were centrifuged, and the supernatants were collected and mixed with 100 μL of ATP detection working solution. Luminescence (RLU) was recorded using a luminometer (Lucetta 2), and ATP concentrations were determined from a standard calibration curve. The results were normalized to the percentage of the blank control group.

1. **Supplementary Tables**

**Table S1.** Formula for the preparation of SeNPs via chemical reduction method.

| **Reagent** | **Concentration** | **Mass** |
| --- | --- | --- |
| Na_2_SeO_3_ | 10 mM | 0.026 g |
| PVA | 20 mg/ml | 0.3 g |
| C_6_H_8_O_6_ | 100 mM | 1.76 g |

**Table S2.** Primers used for RT-PCR.

| **Gene** | **GenBank Accession** | **Sequences** |
| --- | --- | --- |
| GAPDH-mouse | NM_008084 | Forward: AGGTCGGTGTGAACGGATTTG  Reverse: TGTAGACCATGTAGTTGAGGTCA |
| PRDX6 | NM_007453 | Forward: CGCCAGAGTTTGCCAAGAG  Reverse: TCCGTGGGTGTTTCACCATTG |
| GPX4 | NM_008162 | Forward: GATGGAGCCCATTCCTGAACC  Reverse: CCCTGTACTTATCCAGGCAGA |
| SLC7A11 | NM_011990 | Forward: CTGCCTCAACACCTATGACCT  Reverse: GAGAGCAGCAATCAAGAAGGAG |
| HIF-1a | NM_010431 | Forward: ACCTTCATCGGAAACTCCAAAG  Reverse: CTGTTAGGCTGGGAAAAGTTAGG |
| HO-1 | NM_010442 | Forward: AAGCCGAGAATGCTGAGTTCA  Reverse: GCCGTGTAGATATGGTACAAGGA |

1. **Supplementary Figures**

**
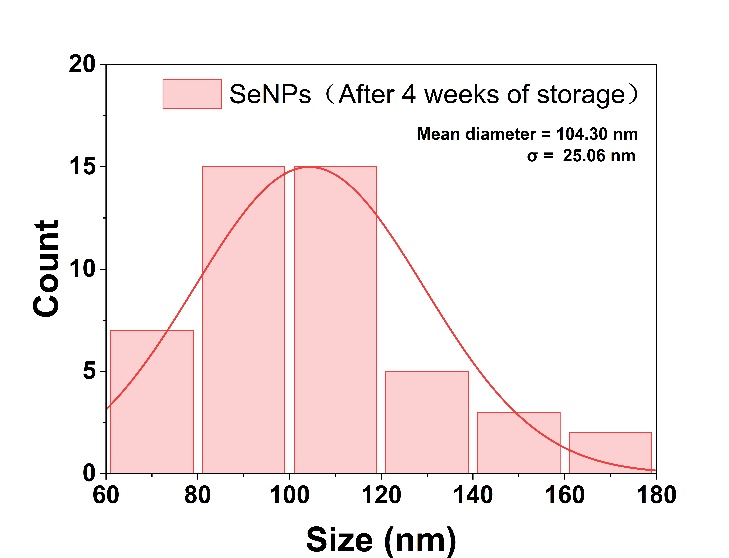
**

**Fig. S1.** Particle size distribution of SeNPs after 4 weeks of storage at room temperature.

**
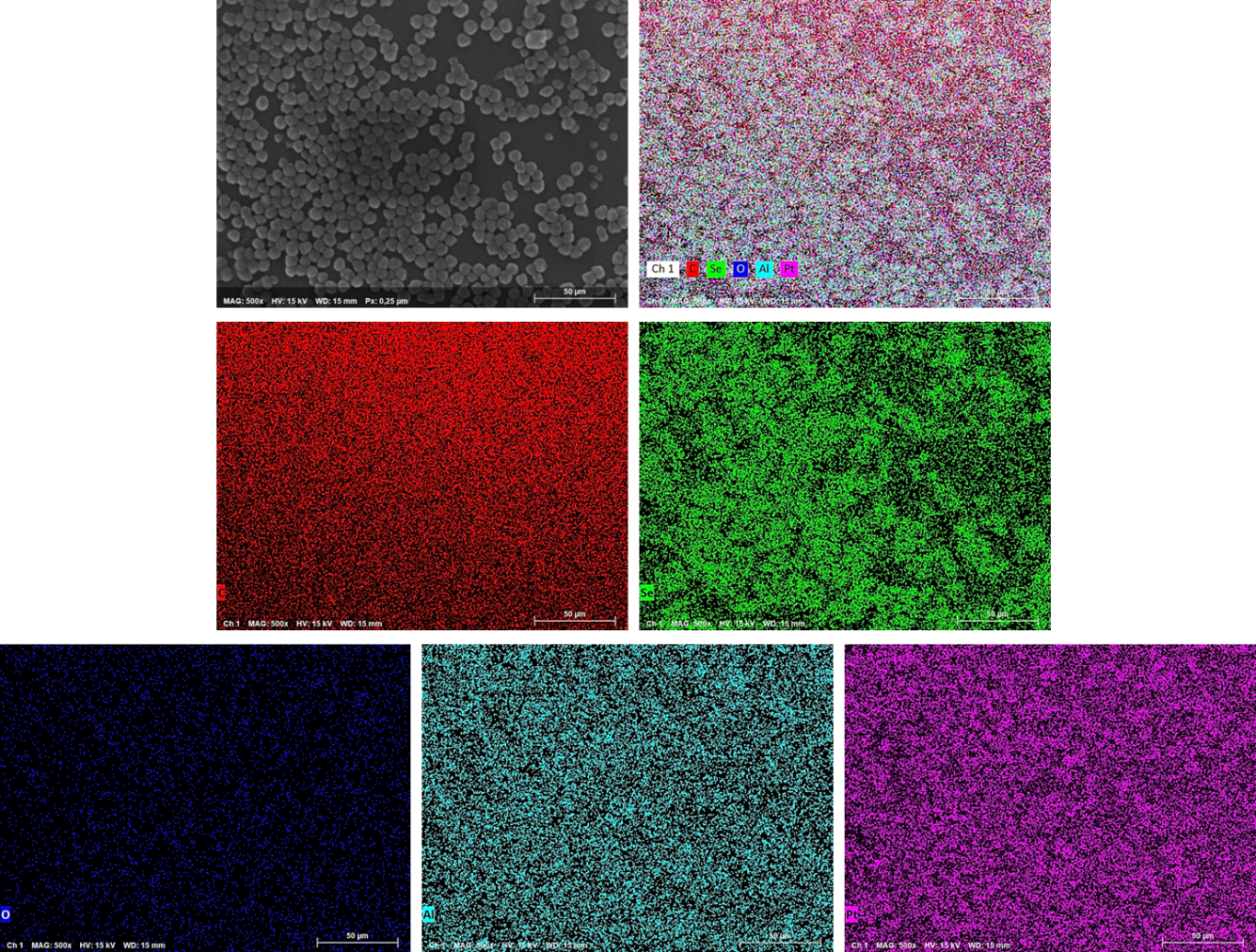
**

**Fig. S2.** EDS analysis of SeNPs in scanning mode.

**
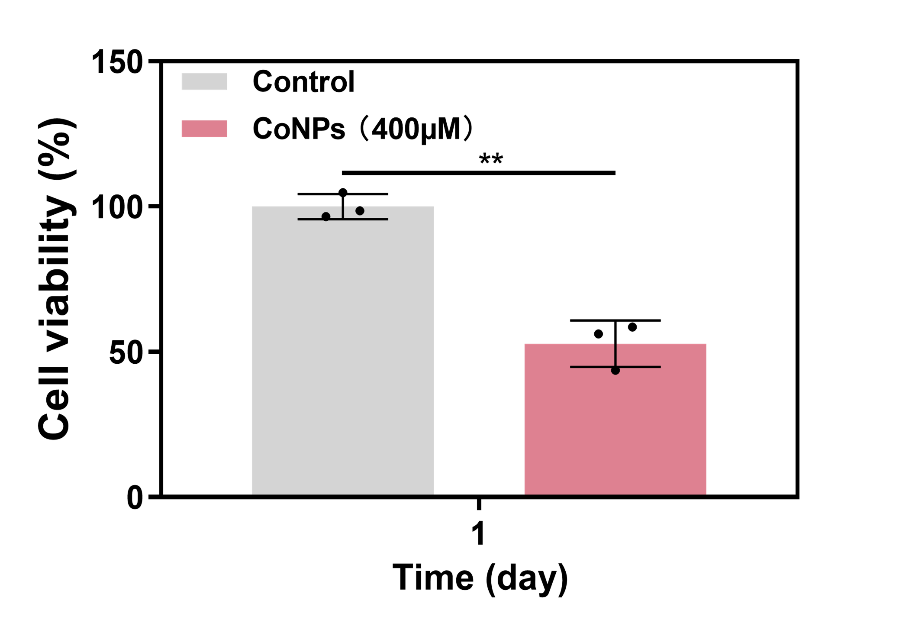
**

**Fig. S3.** Cell viability of CCK-8 assay about BMSCs co-incubated with 400 µM CoNPs or cultured in regular cell culture media for 1 day.

**
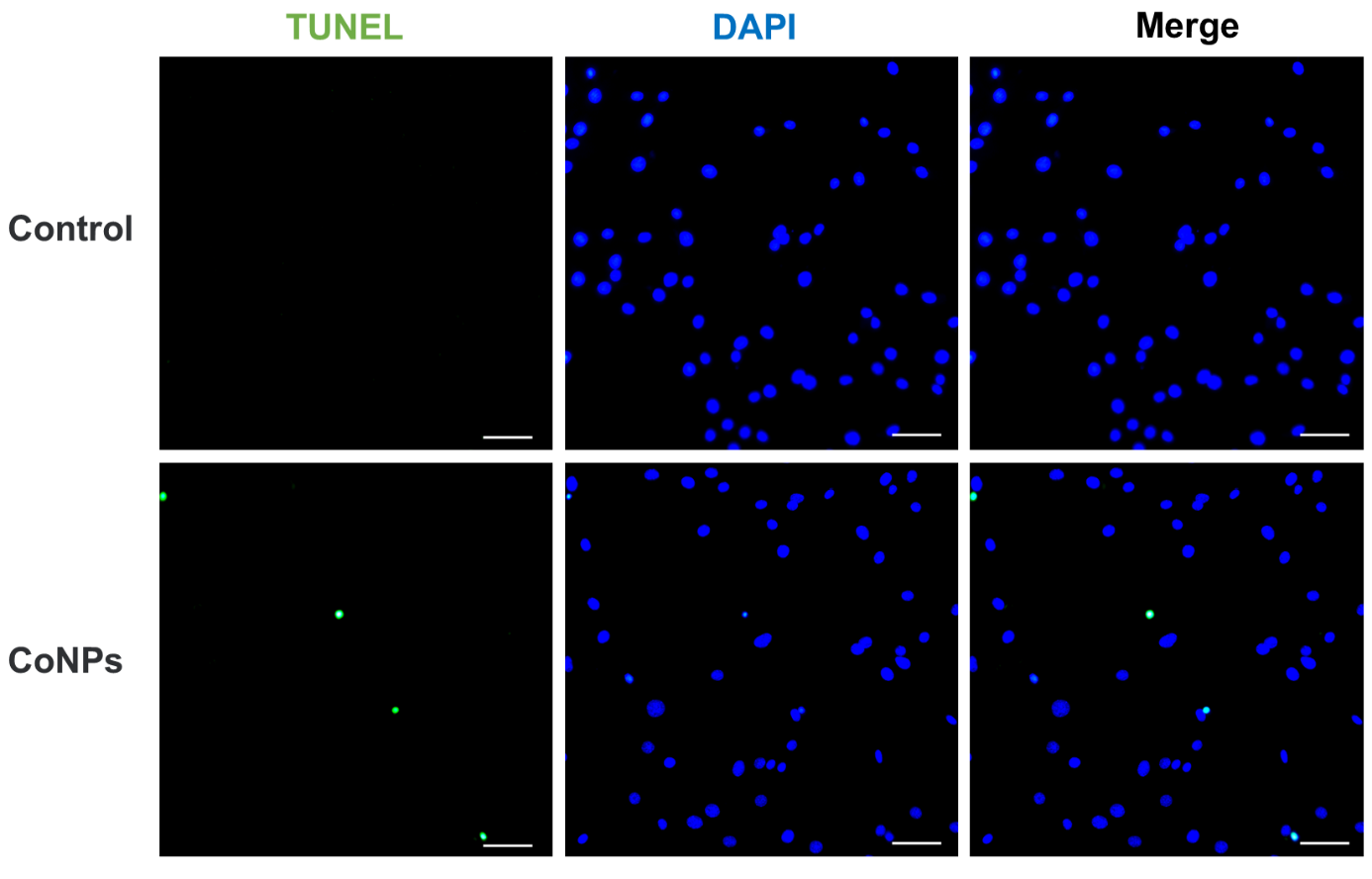
**

**Fig. S4.** Representative TUNEL staining images of BMSCs co-incubated with 400 µM CoNPs for 24 hours. Scale bar = 100 μm.

**
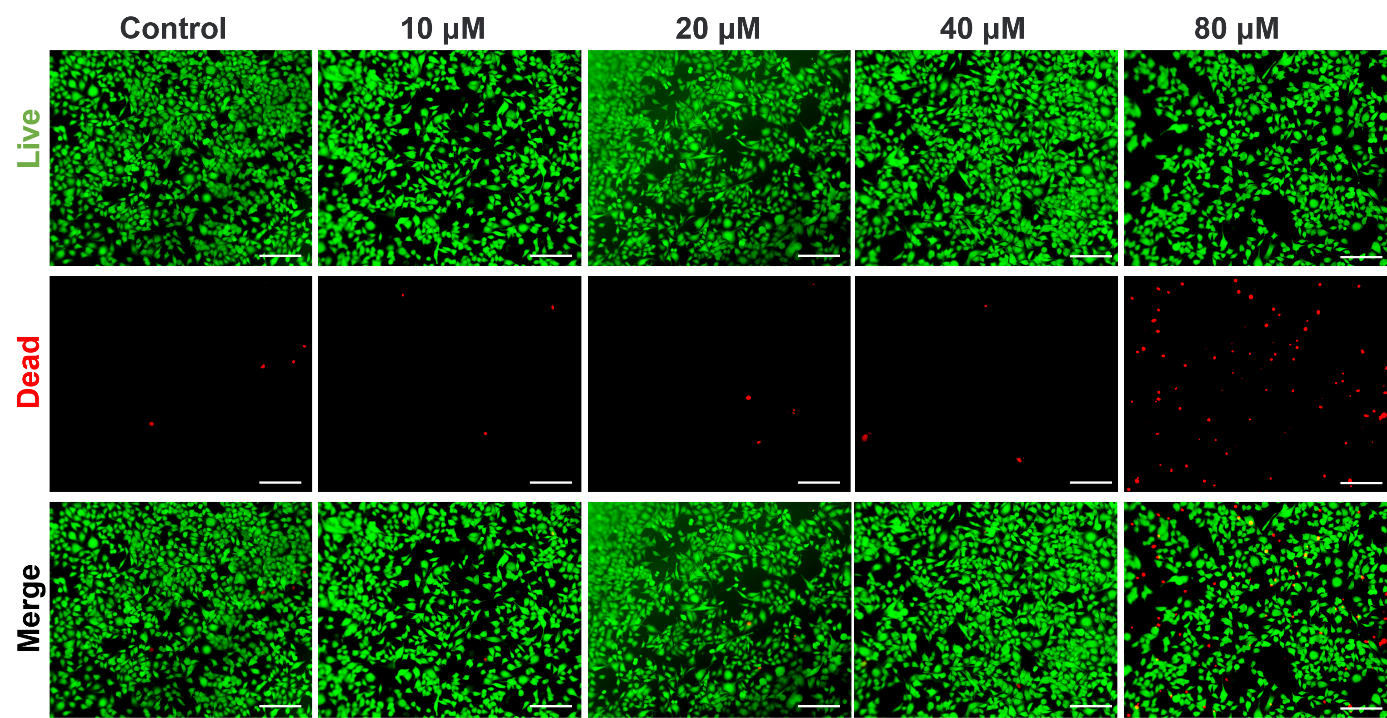
**

**Fig. S5.** Fluorescence micrographs of Live/Dead stained BMSCs after being cultured with different concentrations of SeNPs for 3 days. Scale bar = 100 μm.

**Fig. S6.** Quantification of intracellular total ROS density. (n = 3, ****p*<0.001, *****p*<0.0001)

**Fig. S7.** ATP levels in each group after 24 h of nanoparticles stimulation. The results were normalized to the corresponding values of the control group and expressed as percentages. (n = 3, ***p*<0.01, ****p*<0.001, *****p*<0.0001)

**
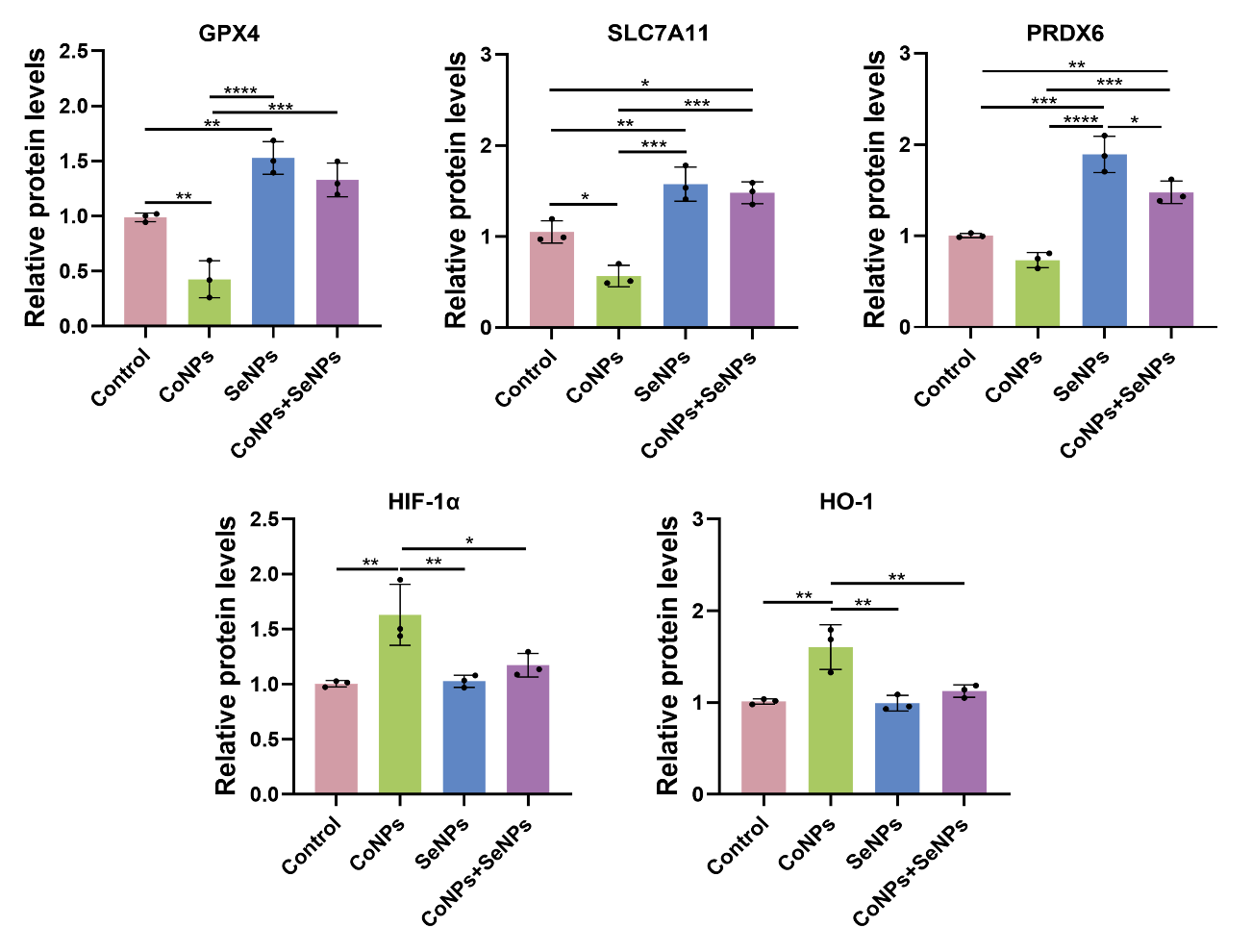
**

**Fig. S8.** The protein expression levels of GPX4, SLC7A11, PRDX6, HIF-1α, and HO-1 were analyzed in BMSCs following treatment with various nanoparticles. The *p*-values by one-way ANOVA are indicated. (n = 3, **p*<0.05, ***p*<0.01, ****p*<0.001, *****p*<0.0001)

**
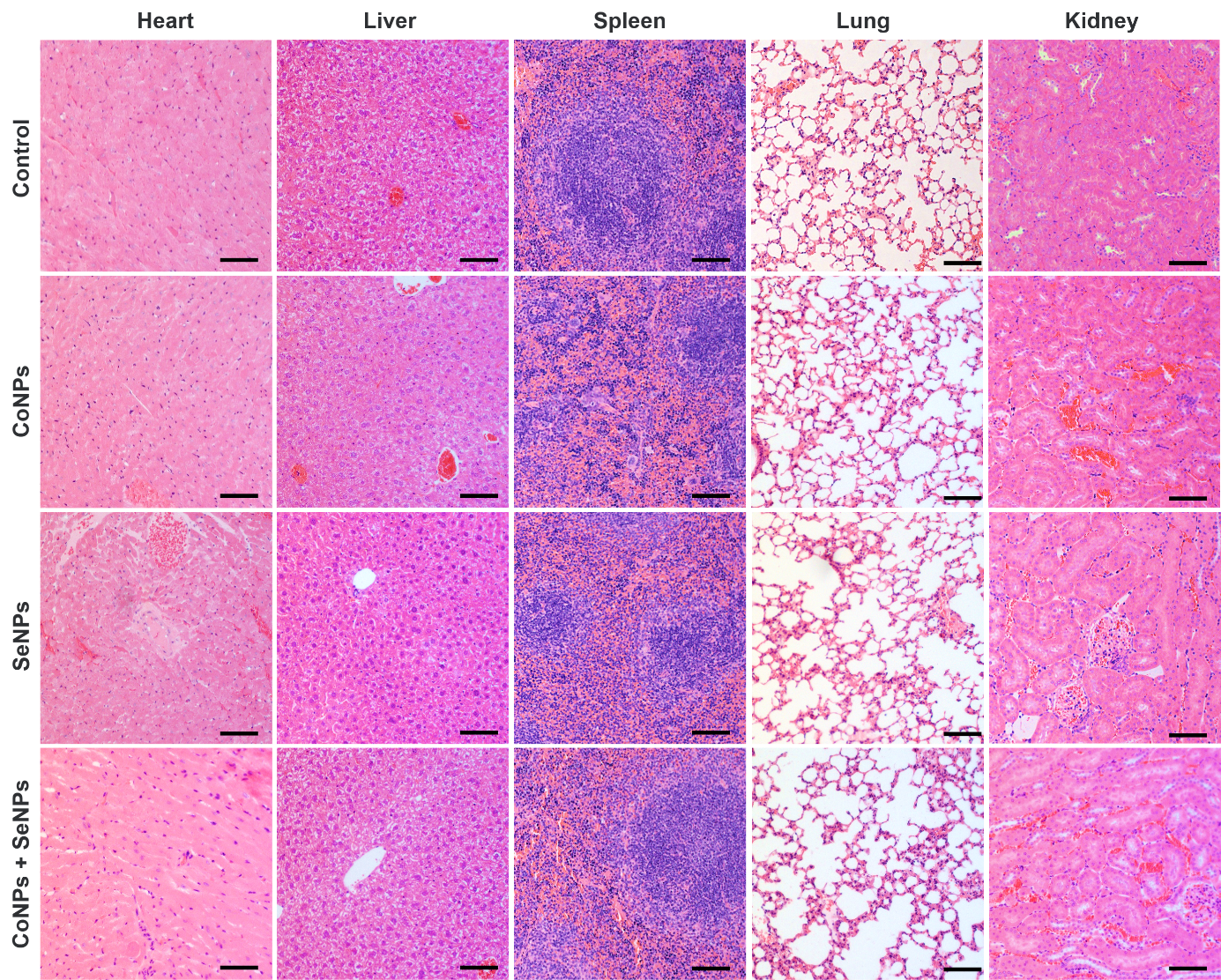
**

**Fig. S9.** Histopathological examination of organ sections (heart, liver, spleen, lung, and kidney) from mice injected with different nanoparticles. Scale bar = 200 μm.

**
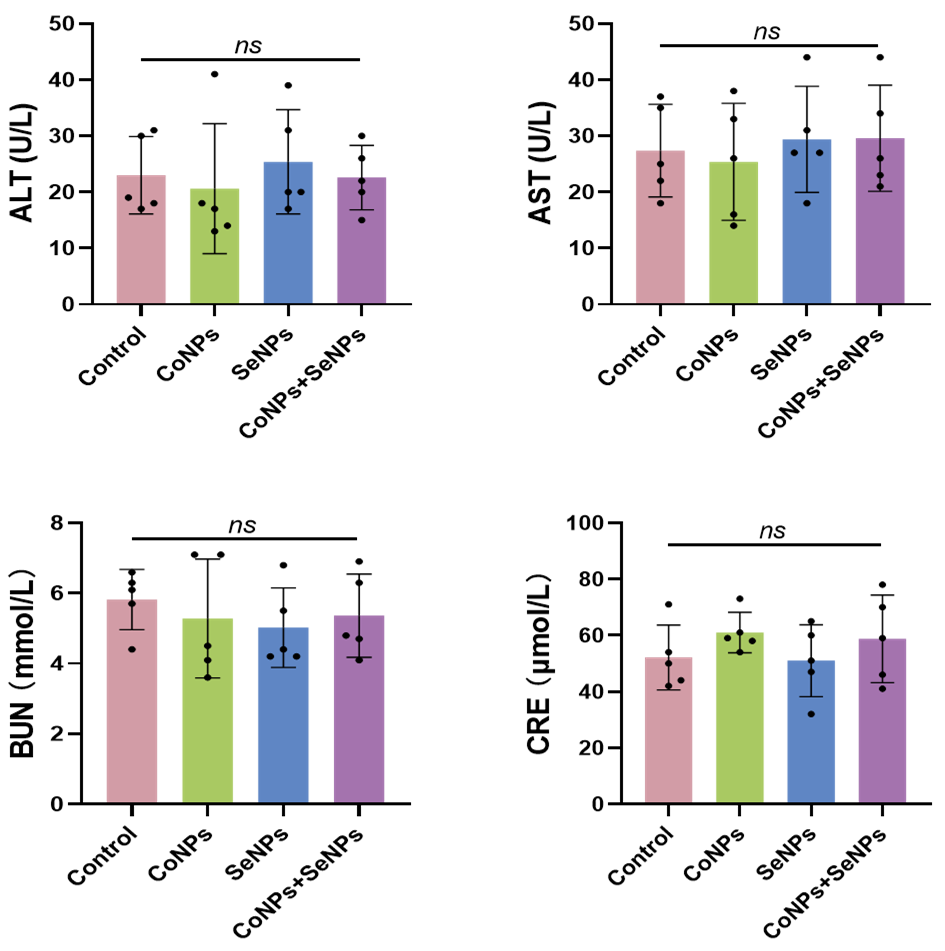
**

**Fig.S10.** Serum biochemical levels (alanine aminotransferase (ALT), aspartate aminotransferase (AST), blood urea nitrogen（BUN）and serum creatinine (CRE)) in mice from different nanoparticle injection groups (n = 5, *ns*: no significance).
